# Influence of a family 29 carbohydrate binding module on the recombinant production of galactose oxidase in *Pichia pastoris*

**DOI:** 10.1016/j.dib.2015.11.032

**Published:** 2015-11-26

**Authors:** Filip Mollerup, Emma Master

**Affiliations:** a Department of Biotechnology and Chemical Technology, Aalto University, 00076 Aalto, Finland; b Department of Chemical Engineering and Applied Chemistry, University of Toronto, 200 College Street, Toronto, Ontario, Canada M5S 3E5

**Keywords:** Galactose oxidase, Carbohydrate binding modules, CBM29, Fermentation, Protein production, Protein purification, Enzyme fusion

## Abstract

Herein, we report the extracellular expression of carbohydrate active fusion enzymes in *Pichia pastoris*. Particularly, CBM29-1-2 from *Piromyces equi* was separately fused to the N- and C-terminus of galactose 6-oxidase (GaO, D-galactose: oxygen 6-oxidoreductase, EC 1.1.13.9, CAZy family AA5) from *Fusarium graminearum,* generating CBM29-GaO and GaO-CBM29, respectively*. P. pastoris* was transformed with expression vectors encoding GaO, CBM29-GaO and GaO-CBM29, and the fusion proteins were expressed in both shake-flask and 2L bioreactor systems. Volumetric production yields and specific GaO activity increased when expression was performed in a bioreactor system compared to shake-flask cultivation. This was observed for both CBM29-GaO and GaO-CBM29, and is consistent with previous reports of GaO expression in *P. pastoris* (Spadiut et al., 2010; Anasontzis et al., 2014) [1], [2]. Fusion of CBM29 to the C-terminal of GaO (GaO-CBM29) resulted in a stable uniform protein at the expected calculated size (107 kDa) when analyzed with SDS-PAGE. By comparison, the expression of the N-terminal fusion protein (CBM29-GaO) was low, and two truncated versions of CBM29-GaO were coexpressed with the full-sized protein. Despite differences in protein yield, the specific GaO activity on galactose was not affected by CBM29 fusion to either the N- or C-terminus of the enzyme. A detailed description of the catalytic and physiochemical properties of CBM29-GaO and GaO-CBM29 is available in the parent publication (Mollerup et al., 2015) [3].

**Specifications Table**

**Table:** 

Subject area	*Biochemistry and Recombinant Protein Production*
More specific subject area	*Recombinant protein expression of fusion proteins in Pichia Pastoris*
Type of data	*Tables and Figures*
How data was acquired	*Through analysis of data from recombinant protein expression*
Data format	*Data is analysed and presented in text*
Experimental factors	*Recominant expression and purification of fusion proteins constructed by separately appending a family 29 carbohydrate binding module to the N- and C-terminus of galactose oxidase*
Experimental features	*Protein expression in shake-flasks and bioreactor systems and chromatographic methods to purify target proteins from cell culture supernatants*
Data source location	*Not applicable*
Data accessibility	*Data is accessible in this article and upon request to the authors*

**Value of the data**

1. These results represent the first production and purification study of galactose oxidase fusions to non-native carbohydrate binding modules, and investigates the impact of CBM positioning on protein recovery.
2. To our knowledge, these expression data present the most active preparation of GaO purified from a *P. pastoris* expression host*,* and simultaneously demonstrate the advantages of using a bioreactor over shake-flask cultivations.
3. Observation of truncated forms of CBM29-GaO, which co-expressed with the full-sized protein. All versions bound efficiently to a Ni-NTA column through a C-terminal His6-tag.
4. Isolation of full-sized CBM29-GaO from its truncated versions by ion-exchange chromatography utilizing slight differences in calculated pI values.

## 1 Data, experimental design, materials and methods

### 1.1 Expression of GaO constructs in shake-flasks

The expression vector, fusion protein sequences, and transformation method are reported in Mollerup et al. [3]. *Pichia pastoris* transformants encoding CBM29-GaO or GaO-CBM29 for extracellular expression were grown overnight in 300 mL buffered minimal glycerol medium (BMGY (w/v): 1% yeast extract, 2% peptone, 100 mM potassium phosphate buffer (pH 6.0), 1.34% YNB, 4×10^−5^% biotin, 1% glycerol) at 30 °C with continuous shaking at 250 rpm. Cells were harvested by centrifugation (1500*g*) at room temperature and suspended in buffered minimal methanol medium containing histidine (BMMH (w/v): 100 mM potassium phosphate buffer (pH 6.0), 1.34% yeast nitrogen base without amino acids (YNB), 4×10^−5%^ biotin, 0.5% methanol, 0.004% histidine) to OD_600_ ~2.0. Cultures were grown at 15 °C for 4 days, and 0.5% methanol was added every 24 h to induce recombinant protein expression. Culture supernatants containing the recombinant protein were harvested by centrifugation at 3000*g* for 30 min and filtered through 0.22 μm PES filter membrane (GE Healthcare – Life Sciences) before purification (see below).

The activity of wild-type GaO and GaO fusions was measured using the previously described chromogenic ABTS (2,2′-azino-bis(3-ethylbenzothiazoline-6-sulfonic acid)) assay [4]. The standard reaction mixture (final volume: 200 μL) contained 7 U/mL horseradish peroxidase, 2 mM ABTS, and 300 mM galactose in 50 mM sodium phosphate buffer (pH 7.0); reactions were then initiated by adding 5 μL of the enzyme sample diluted to accurately measure initial reaction rates. Reactions were monitored at 420 nm at 30 °C for 3 min. Hydrogen peroxide (from 5×10^−4^ to 5×10^−2^ μmole) was used to generate a standard curve. Each reaction was performed in triplicate, at minimum.

**Fig. 1:**
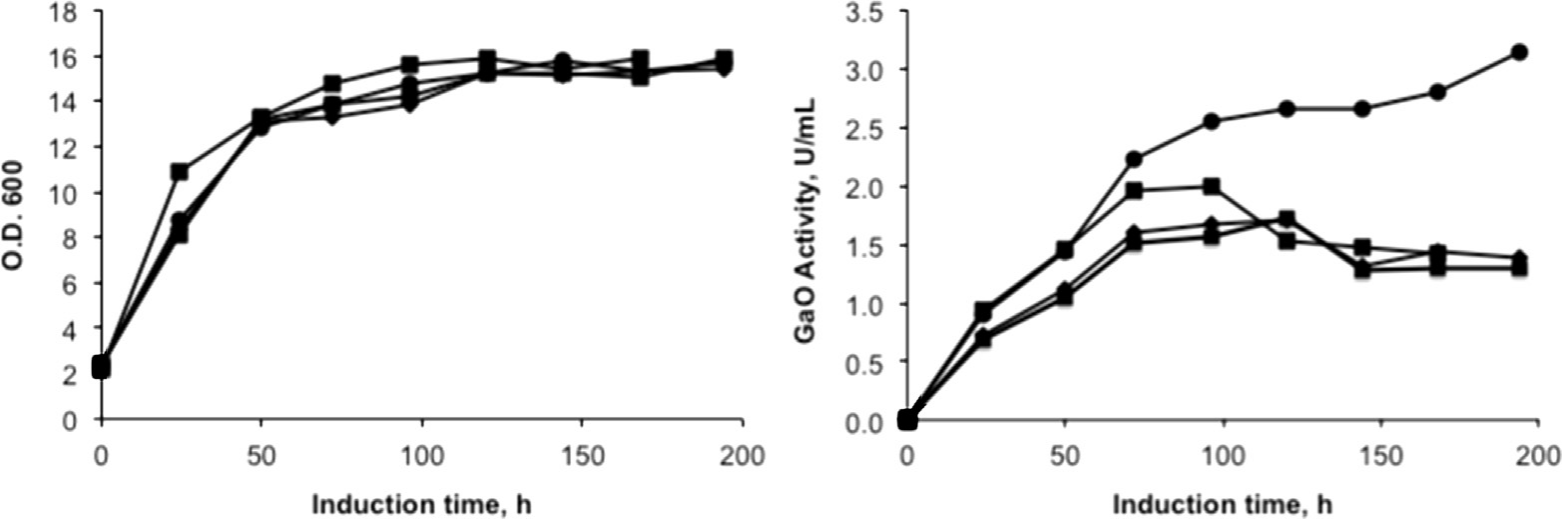
Cell density and volumetric galactose oxidase activity of GaO-CBM29 in shake-flasks cultivations after first induction**.** ■ Standard cultivation (pre-cultivation at 30 °C and induction at 15 °C); ♦ standard cultivation medium plus 10 μM leupeptin in the induction medium, **×** pre-cultivation at 15 °C prior to induction; **●** standard cultivation plus addition of 0.5 mM copper sulfate to the induction medium.

Similar to studies reporting GaO production in *P. pastoris* [1], the addition of 0.5 mM copper sulfate to *P. pastoris* shake-flask cultivations increased GaO-CBM29 activity in the cultivation medium by more than 2 times (Fig. 1).

### 1.2 Expression of GaO constructs in a bioreactor system

All fermentations were performed using a Biostat B Plus bioreactor (Sartorius) equipped with pH and oxygen probes from Hamilton and connected to a MFCSwin process control system (BBI B. Braun Biotech International). Cultivation conditions were based on Pichia Fermentation Process Guidelines provided by Invitrogen, with minor modifications. Briefly, a 100 mL inoculum of *P. pastoris* KM71H (Mut^S^) expressing wild-type GaO and *P. pastoris* SMD1168H (Mut^+^) expressing GaO/CBM29 fusions were cultivated in YPD at 30 °C to an OD_600_ between 2 and 6. Cells were harvested by centrifugation (3000*g*, 5 min) and then suspended in basal salts medium (26.6 mL 85% phosphoric acid, 0.93 g calcium sulfate, 18.2 g potassium sulfate, 14.9 g magnesium sulfate-heptahydrate, 4.13 g potassium hydroxide, 40 g glycerol per liter). The initial fermentation medium comprised 1 L basal salts medium containing 4% (w/v) glycerol and 0.435% (v/v) PTM_1_ trace salts (6.0 g cupric sulfate pentahydrate, 0.08 g sodium iodide, 3.0 g manganese sulfate monohydrate, 0.2 g sodium molybdate dihydrate, 0.02 g boric acid, 0.5 g cobalt chloride, 20.0 g zinc chloride, 65.0 g ferrous chloride heptahydrate, 0.2 g biotin, 5.0 ml sulfuric per liter). The pH was controlled to pH 6.0 by automatic addition of 15% (w/v) ammonium hydroxide, which also served as the sole nitrogen source during cultivation. The dissolved oxygen concentration was maintained above 40% by automatic cascade stirring and gas flow feedback-control, using the proportional–integral–derivative (PID) controller function in the bioreactor. Specifically, the stirrer speed was first increased from 300 to 1200 rpm and then the airflow was increased from 0.5 to 3.0 L min^−1^ until maximum oxygen transfer capacity of the reactor was reached. Antifoam (Struktol J 647) was added automatically as required. Upon depletion of glycerol from the medium the glycerol fed-batch phase was initiated manually by feeding a 50% (w/v) glycerol mix, with 1.2% (v/v) PTM_1_ trace salts, to the reactor at initially 15 mL h^−1^, and stepwise increased until the maximum oxygen transfer capacity was reached while still maintaining a substrate-limited growth rate and dissolved oxygen concentration between 25% and 35% (though the feed rate was not further increased once reaching a maximum of 25 mL h^−1^).

For induction, the temperature was lowered to 20 °C, the glycerol feed was stopped, and the dissolved oxygen concentration was allowed to increase as residual glycerol was consumed by the cultures. To express fusion constructs from *P. pastoris* SMD1168H and GaO from *P. pastoris* KM71H, methanol with 1.2% (v/v) PTM_1_ trace salts was added stepwise from 0.5%, to 1.0% and then 2.0%, while allowing the dissolved oxygen to stabilize prior to the next methanol addition. In this way, cultures were presumably acclimatized to methanol metabolism prior to feeding with methanol at a flow rate of 7.0 mL h^−1^ for SMD1168 Mut^+^ strains and 2.7 mL h^−1^ for the KM71H Mut^S^ strain. For all cultivations, DO was monitored to ensure that oxygen consumption was limited by methanol metabolism.

Production of the fusion constructs along with wild-type GaO in the bioreactor system allowed better control of temperature, aeration, and methanol feed. Accordingly, the yield of purified CBM29-GaO and GaO-CBM29 were more than 12 and 6 times higher than from shake flasks; corresponding specific activities also increased by 20% and 37%, respectively (Table 1), potentially due to impacts of growth condition on functional folding and post-translational processing, and the presence of copper(II) in the medium. These trends are consistent with a recent study aimed at optimizing GaO production in bioreactor systems [2], and so these samples were used for all subsequent analysis.

### 1.3 Purification of recombinant GaO

Protein in culture supernatants from shake-flaks cultivations was precipitated using ammonium sulfate (up to 70 (w/v)% of saturation at 4 °C). Protein pellets were recovered by centrifugation, dissolved in a minimal volume of 50 mM sodium phosphate buffer (pH 7.0), and then filtered through a Sterivex-GP 0.22 μm PES filter unit (Millipore). The resulting concentrates were separately incubated overnight at 4 °C with 20 mL of 50% Ni-NTA resin (Qiagen) equilibrated in the same buffer. Each Ni-NTA resin was then transferred into an empty XK-16/10 column (GE Life Sciences) resulting in a packed bead volume of 10 mL. Protein with unspecific binding was washed from the Ni-NTA resin using 10 column volumes (CV) of sodium phosphate buffer (pH 7.0). Fractions (5 mL) were eluted at a flow rate of 0.5 cm/min using a linear gradient of 0–100% Ni-NTA elution buffer (400 mM imidazole in 50 mM sodium phosphate buffer (pH 7.0)) over 10 CV. All chromatographic steps were performed at room temperature using an Äkta chromatography system (GE Life Sciences). Fractions containing purified GaO*-*CBM29 were then concentrated and exchanged into 50 mM sodium phosphate buffer (pH 7.0) using a Vivaspin 20 unit (30,000 MWCO; GE Healthcare Life Sciences).

**Fig. 2:**
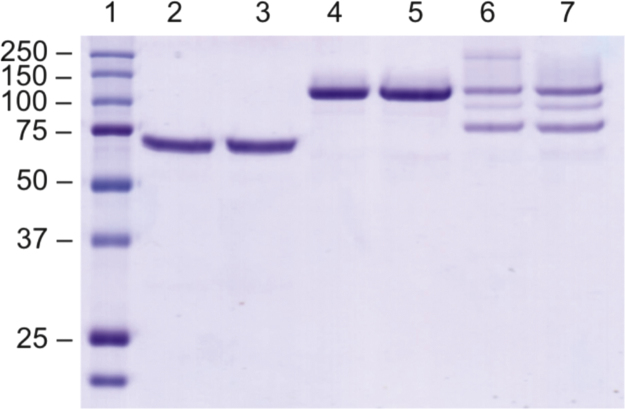
Electrophoretic molecular weight of purified enzymes isolated from bioreactor cultivations. Lanes, 1: Precision Plus PMWL, 2: GaO (untreated), 3: GaO (treated with CuSO_4_), 4: GaO-CBM29 (untreated), 5: GaO-CBM29 (treated with CuSO_4_), 6: partially purified CBM29-GaO (untreated), 7: partially purified CBM29-GaOx (treated with CuSO_4_). Treated samples were incubated for 120 h with CuSO_4_ at 4 °C. The complete assessment of CuSO_4_ on the recombinant enzymes is shown in Fig. 4 and Table 2.

**Fig. 3:**
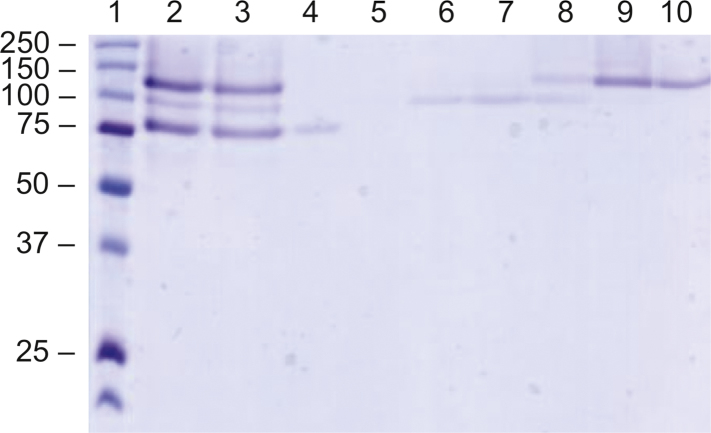
Ion-exchange chromatography of CBM29-GaO to isolate the 107 kDa band from truncated versions coeluted from NiNTA purification. Lanes, 1: Precision Plus PMWL, 2: CBM29-GaO from lane 7 in Fig. 2 and 3: CBM29-GaO adjusted to 50 mM sodium phosphate (pH 7.0), 4: flow-through, 5–9 elutions with increasing sodium chloride concentration, 10: CBM29-GaO desalted in 50 mM sodium phosphate (pH 7.5).

Purification of wild-type GaO and fusion constructs from bioreactor cultivations were modified as follows. At the end of each fermentation, the pH was automatically adjusted to 7.5 for purification using 4 M sodium hydroxide. This also resulted in precipitation of residual fermentation medium salts in the medium. The supernatant was then harvested by centrifugation at 13,680*g* for 60 min at 4 °C, adjusted to 1 M ammonium sulfate by dilution with a 4 M stock, and then filtered through a 0.22 μm PES filter unit. The pH was checked again before the protein solution was subsequently loaded onto a 10 mL phenyl sepharose FF column equilibrated to 50 mM sodium phosphate containing 1.0 M ammonium sulfate, with a flow rate of 2.5 cm/min. Weakly bound protein was removed using 5 CV of the equilibration buffer, and then 50 mL fractions were eluted at a flow rate of 2.5 cm/min using a linear gradient of 1.0 M ammonium sulfate to 50 mM sodium phosphate buffer (pH 7.5) over 10 CVs. As above, all chromatographic steps were performed at room temperature using an Äkta chromatography system. CBM29-GaO and GaO-CBM29 were further purified using Ni-NTA affinity resin as described above. While this yielded a single protein band as visualized by SDS-PAGE that also corresponded to the expected size for GaO-CBM29, SDS-PAGE assessment of CBM29-GaO fractions showed that two proteins co-eluted from the IMAC column (Fig. 2). To further separate CBM29-GaO from putative truncated forms, fractions containing CBM29-GaO were desalted and adjusted to pH 7.0 in 50 mM sodium phosphate using a Vivaspin 20 (MWCO 30.000). The sample was loaded on a 1 mL DEAE-sepharose column (HiTrap DEAE FF, GE Life Sciences) with a flow rate of 0.5 cm/min and eluted by an increasing concentration of sodium chloride (0–1 M), to then reach over 95% purity as assessed by SDS-PAGE (Fig. 3).

In all cases, protein samples were transferred to 50 mM sodium phosphate buffer (pH 7.5) with continuous dilution and concentration using a Vivaspin (MWCO 30.000 Da) before using the Bradford method (Bio-Rad Laboratories, USA) to measure protein concentration and SDS-PAGE to confirm enzyme purity. Purified samples were then flash frozen with liquid nitrogen and stored at −80 °C.

The molecular mass of recombinant CBM29-GaO and GaO-CBM29 estimated by SDS-PAGE was 107 kDa, which is consistent with the predicted molecular mass of these enzymes (107.3 kDa) (Fig. 2). Correspondingly, the yield of purified CBM29-GaO and GaO-CBM29 from shake-flask cultivations induced at 15 °C was 0.65 mg/L and 1.5 mg/L, respectively (Table 1). Enzyme identities were verified by mass spectrometric analysis of tryptic fragments (Biomedicum, University of Helsinki).

### 1.4 GaO activation

**Fig. 4:**
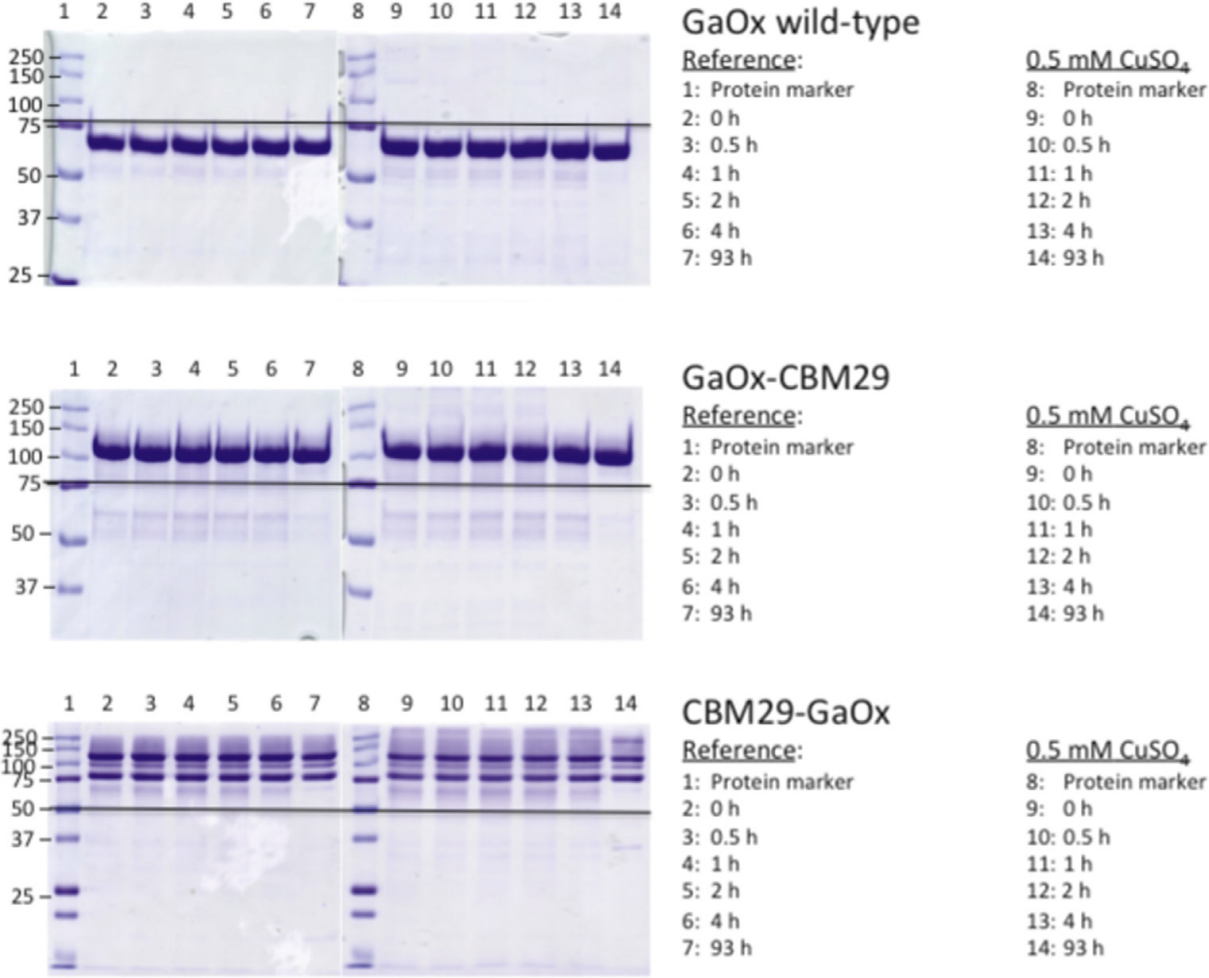
Electrophoretic molecular weight of purified enzymes following incubation in 0.5 mM CuSO_4_ at 4 °C. Sample identities are indicated by the inserted legends.

**Fig. 5:**
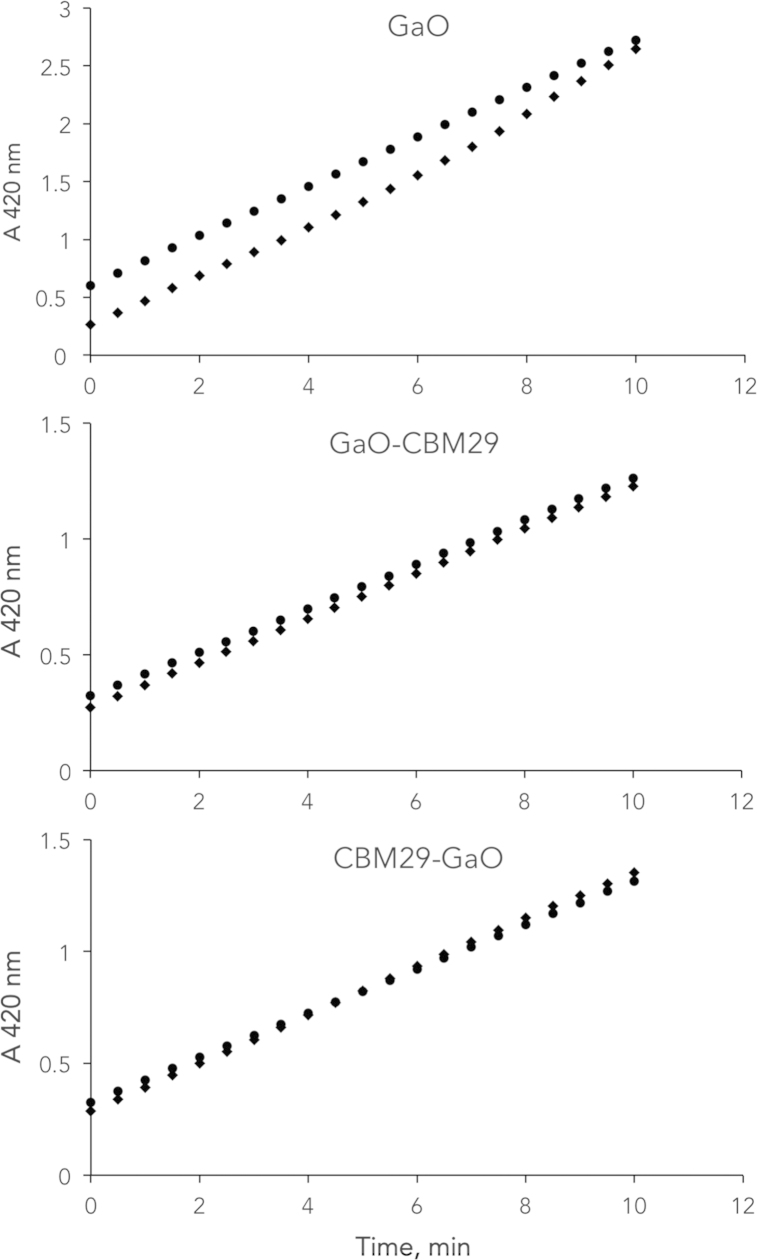
Impact of potassium ferricyanide on GaO activity using enzymes purified from bioreactor cultivations. Approximately 7.5 μg (0.5 mg/mL) of protein was incubated for 15 min. at room temperature in presence or absence of 0.46 mM K_4_Fe(CN)_6_. The samples where diluted 1000 times prior activity determination. ● samples in 50 mM sodium phosphate alone, ♦ samples containing 0.46 mM K_4_Fe(CN)_6_.

To confirm full activation of GaO, purified wild-type GaO and GaO fusions from bioreactor cultivations were treated with copper sulfate and potassium ferricyanide as previously described [4], [5], [6]. Specifically for this study, 0.15 mg/mL of purified enzymes were incubated in 0.5 mM copper sulfate for up to 120 h (Fig. 2) at 4 °C. After 0.5, 1, 2, 4, 18, 93 and 120 h of incubation, samples were recovered for SDS-PAGE analyses (Fig. 2, Fig. 4) and activity measurement (Table 2) using the standard activity assay. Purified enzymes were also separately treated with 0.46 mM potassium ferricyanide at room temperature for 10 min and then analyzed for activity (Fig. 5). In all cases, enzymes were diluted to 0.15 mg/mL prior activity determination, and enzymes incubated in reaction buffer alone were used as references.

Whereas adding copper sulfate to shake flask cultivations doubled GaO activity measured in the cultivation medium (Fig. 1), incubation of purified GaO-CBM29 and CBM29-GaO from bioreactor cultivations with 0.5 mM copper (II) sulfate under aerobic conditions did not further increase the specific activity of these enzymes (Table 2), or cause corresponding protein bands to shift when observed by SDS-PAGE (Fig. 4). Similarly, treatment with 0.46 mM potassium ferricyanide did not increase enzyme activities, suggesting that the purified wild-type GaO and fusion proteins were in the fully oxidized and active state [Cu^2+^-Tyr^●^-Cys] (Fig. 5).

## Figures and Tables

**Table 1:** Yield and activity of purified wild-type galactose oxidase and CBM29 fusions.

**Enzyme**	**Molecular weight (kDa)**	**Production host**	**Shake flask cultivation**		**Fermentation**	
**Yield (mg/L)**^a^	**Specific activity**^b^**(Units/mg)**	**Yield (mg/L)**	**Specific activity**^b^**(Units/mg)**
GaO (wt)	*68*	*P. pastoris* KM71	ND	ND	104.2	516±25
GaO-CBM29	*107*	*P. pastoris* SMD1168	1.5	355.4±23	13.6	487±10
CBM29-GaO	*107*	*P. pastoris* SMD1168	0.64	363.8±32	1.4^c^	438±29

a Yields refer to mg of purified enzyme (>95%) per liter of cultivation medium.

b Specific activity of fully mature and active GaO, GaO-CBM29 and CBM29-GaO (Fig. 3 lane 10) was measured using 300 mM galactose; *n*=3.

c Refers to final yield of the 107 kDa protein (after EIX purification (Fig. 3)). Purified yield of all the recombinant versions of CBM29-GaO (70, 90 and 107 kDa) was 3.4 mg/mL.

**Table 2:** Relative enzyme activity following incubation at 4 °C in 50 mM sodium phosphate with or without 0.5 mM CuSO_4_. The activity of reaction mixtures at 0 h that were subsequently amended with CuSO_4_ were defined as 100%.

**Incubation time (h)**			**Relative activity (%)**			
**GaO**		**GaO-CBM29**		**CBM29-GaO**	
CuSO_4_	Buffer	CuSO_4_	Buffer	CuSO_4_	Buffer
0	100	90	100	101	100	97
0.5	107	109	99	92	69	75
1	101	96	89	99	68	81
2	104	97	83	94	72	79
4	109	95	84	100	81	85
18	116	97	84	99	85	80
93	105	85	79	82	54	60
